# The Intrahepatic Expression and Distribution of BTLA and its Ligand HVEM in patients with HBV-related acute-on-chronic liver failure

**DOI:** 10.1186/1746-1596-7-142

**Published:** 2012-10-15

**Authors:** Huan Xu, Dayan Cao, Guoning Guo, Zhihua Ruan, Yuzhang Wu, Yongwen Chen

**Affiliations:** 1 Institute of Immunology, PLA, Third Military Medical University, Chongqing, 400038, People’s Republic of China; 2 Undergraduate Administration Office, Third Military Medical University, Chongqing, 400038, People’s Republic of China; 3 Department of Emergency, South-West Hospital, PLA, Third Military Medical University, Chongqing, 400038, People’s Republic of China; 4 Department of Oncology, South-West Hospital, PLA, Third Military Medical University, Chongqing, 400038, People’s Republic of China

**Keywords:** BTLA, HBV-ACLF, HVEM, Immunohistochemistry, B7 superfamily

## Abstract

**Objective:**

It has been demonstrated that signals from the inhibitory receptor B and T lymphocyte attenuator (BTLA) are involved in regulating the pathogenesis of infectious diseases. However, the expression and anatomical distribution of BTLA and its ligand, the herpes virus entry mediator (HVEM), have not yet been determined in cases of HBV-related acute-on-chronic liver failure (HBV-ACLF) patients.

**Methods:**

In this study, the expression of BTLA and HVEM in liver tissues from HBV-ACLF, chronic hepatitis B (CHB) patients and healthy individuals was analyzed by immunohistochemistry.

**Results:**

The results of this analysis demonstrated that both molecules were observed in the HBV-ACLF samples and that their expression was chiefly in the infiltrating inflammatory cells and the damaged bile ducts. However, they were absent in liver sections from CHB patients and healthy controls. Immunofluorescence double-staining indicated that BTLA was found on CK-18^+^ epithelial cells, CD31^+^ endothelial cells, CD68^+^ macrophages, CD56^+^ NK cells, CD16^+^ monocytes, CD3^+^ , CD8^+^ T cells, and Foxp3^+^ regulatory T cells (Treg). By contrast, HVEM expression was restricted to CK18^+^ epithelial cells and CD68^+^ macrophages. Moreover, the expression of several members of the B7 superfamily, including PD-L1, PD-L2, B7-H3 and B7-H4, was also detected in these liver tissues, and these proteins were co-expressed with HVEM. Interestingly, the expression of fibrinogen-like protein 2 (FGL2), a virus-induced procoagulant molecule, was also found in liver sections from HBV-ACLF, this molecule also co-expresses with BTLA and HVEM.

**Conclusions:**

These results suggest that BTLA-HVEM signaling is likely to affect the pathogenesis of HBV-ACLF, a clear understanding of the functional roles of these proteins should further elucidate the disease process.

**Virtual slides:**

The virtual slide(s) for this article can be found here: http://www.diagnosticpathology.diagnomx.eu/vs/8080806838149123

## Introduction

Hepatitis B virus (HBV) infection causes a spectrum of diseases, such as chronic hepatitis B (CHB), liver cirrhosis, primary hepatocellular carcinoma, and acute-on-chronic liver failure (ACLF) [1]. HBV-ACLF is a clinical syndrome that is defined as acute hepatic insult with either diagnosed or undiagnosed chronic liver disease [2,3]. The hallmark of HBV-ACLF is the extreme rapidity of the necromicroinflammatory process, which results in hepatocellular necrosis within several weeks [4]. Currently, liver transplantation is the best method for clinical treatment of this disease. However, the mortality of HBV-ACLF remains very high. It is essential to identify more specific markers for the diagnosis of this disease.

B and T lymphocyte attenuator (BTLA) is a member of the CD28 superfamily [5]. BTLA-deficient (BTLA^−/−^) mice are more sensitive to experimental autoimmune encephalomyelitis (EAE) [5], allergic airway inflammation [6], autoimmune hepatitis [7] and MHC-mismatched allograft rejection [8] than normal individuals, indicating that this receptor plays an essential role in maintaining peripheral tolerance. In infectious disease models, for instance, it has been found that an interruption of BTLA signaling causes the augmentation of the virus-specific cytotoxic lymphocyte (CTL) response, promoting the early clearance of both *Listeria* bacteria and CMV infection [9,10]. These studies generally indicate that BTLA appears to be a negative regulator. Herpes virus entry mediator (HVEM), an immunoregulatory molecule that belongs to the tumor necrosis factor (TNF) receptor superfamily, has been identified as the potential ‘ligand’ for BTLA [11]. The cross-linking of BTLA with HVEM results in the phosphorylation of the tyrosines on BTLA and the recruitment of phosphatases, including SHP-1 and SHP-2, to the ITIM motif in the cytoplasmic region of BTLA, this response reduces TCR signaling and eventually diminishes T cell activation [12].

Our previous work has demonstrated that BTLA actively participates in the pathogenesis of fulminant hepatitis that is caused by infection with murine hepatitis virus strain 3 (MHV-3) [13]. However, uncertainty remains regarding the expression and anatomical distribution of BTLA and HVEM in livers from HBV-ACLF patients. In this study, we used immunohistochemistry to analyze the expression of both proteins in liver biopsies from HBV-ACLF patients and further assessed the phenotypes *via* immunofluorescence double staining.

## Materials and methods

### Patients

Four samples from autopsies of HBV-ACLF patients, 16 samples from liver biopsies of CHB patients, and 5 samples of liver tissue from normal individuals were used in this study. All of the CHB patients were seropositive for hepatitis B surface Ag (HBs) and hepatitis B e Ag (HBe) for at least 12 months and were negative for antibodies against the human immunodeficiency virus, hepatitis C virus, and hepatitis D virus, additional parameters for these CHB patients have been reported in our previous work [14,15]. All of the patients were admitted to the Center of Infectious Disease, Southwest Hospital (Chongqing 400038, People’s Republic of China) from June 2006 to January 2009. None of the CHB patients were treated for chronic HBV infection for 6 months prior to the liver sampling process. Sections of liver tissue were scored in a blinded fashion to measure their histological diagnoses. The study protocol was approved by the Ethics Committee of the Third Military Medical University review board.

### Immunohistochemistry

The protocol that was used for immunohistochemistry was in accordance with previously published procedures, with slight modifications [14]. Briefly, paraffin-embedded tissue blocks were cut into 2~3 μm sections and mounted on poly-l-lysine-charged glass slides. After the sections were dewaxed and rehydrated, antigen retrieval was performed by microwaving these sections in 10 mM citrate buffer (pH 6.0). The sections were cooled to room temperature (RT), and the endogenous peroxidase was blocked by incubation with a solution of 0.5% hydrogen peroxide (H_2_O_2_) in 50% methanol for 1 h. The sections were then incubated in 3% BSA plus 0.1% Nonidet P-40 in PBS for 1 h at RT to block nonspecific binding. The sections were then incubated overnight at 4°C with primary anti-BTLA (1:100, rabbit IgG; Santa Cruz, San Diego, CA, USA), anti-HBs (1:100, rabbit IgG; Abcam, Cambridge, MA, USA), anti-CD68 (1:100, clone: 3F103; Santa Cruz), anti-CD16 (1:100, clone: 2Q1240; Santa Cruz), anti-CD3 (1:50, clone: F7.2.38; Dako, Copenhagen, Denmark) or anti-fibrinogen (1:100; Dako) antibodies that had been diluted in 1% BSA. After the sections were washed, they were incubated with the corresponding secondary antibodies for 1 h at RT. The Vectastain ABC kit (Vector Laboratories, Burlingame, CA, USA) was used to implement the avidin–biotin complex approach in accordance with the manufacturer’s instructions. Sections that were incubated with isotype-matched, concentration-matched immunoglobulin without primary antibodies were used as isotype controls. Peroxidase activity was visualized with the DAB Elite kit (K3465, Dako), and the brown coloration of tissues represented positive staining. The sections were lightly counterstained with hematoxylin, dehydrated through an ethanol series to xylene, and mounted. Finally, the sample sections were viewed using a light microscope (Zeiss Axioplan 2, Berlin, Germany).

### Immunofluorescence double staining

For immunofluorescence double staining, sections were incubated with primary anti-BTLA and anti-HVEM antibodies at 4°C overnight. After the sections were washed with PBS (5 min/wash × 3 washes), they were incubated with Alexa Fluor® 555-conjugated goat anti-mouse/rabbit IgG antibodies (Invitrogen, San Diego, CA, USA) for 1 h. Subsequently, the sections were further incubated with anti-CD3, anti-CD8 (1:50, clone: C8/144B; Dako, Denmark), anti-CD56 (1:50; Santa Cruz), anti-B7-H1, (1:100, clone: 29E.2A3; kindly provided by Dr. Gordon J. Freeman (Department of Medical Oncology, Dana-Farber Cancer Institute, Harvard Medical School, USA)), anti-B7-DC (either 1:50, polyclonal goat IgG; R&D Systems or 1:100, clone: 24F.7G12; kindly provided by Dr. Gordon J Freeman), anti-PD-1 (2 μg/ml, AF1086; R&D Systems), anti-CD68, anti-FGL2 (1:100, clone: 4H5; Santa Cruz), anti-CD31 (1: 50; Santa Cruz), anti-CK-18 (1: 200; Santa Cruz), or anti-FGL2 (1:100; Santa Cruz) antibodies at 4°C overnight and incubated with Alexa Fluor® 488-conjugated goat anti-mouse/rabbit IgG1 antibodies (Invitrogen) for an additional 1 h. Finally, the sections were incubated with 1 μg/ml DAPI (Sigma, St. Louis, MO, USA) for 10 min to stain the nuclei. Sections incubated with the appropriate isotype control primary antibodies and fluorescently labeled secondary antibodies were used as negative controls. The results were analyzed using fluorescence microscopy (Zeiss Axioplan 2).

## Results

### The pathologic characteristics of liver tissue from HBV-ACLF

**Figure 1:**
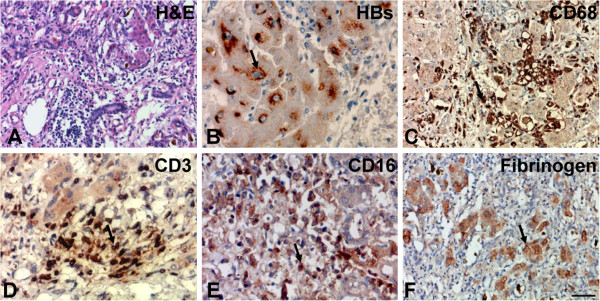
**The morphology of liver tissue from patients with HBV-related acute-on-chronic liver failure (HBV-ACLF).** (**A**) HE staining demonstrated that liver tissue sections from HBV-ACLF patients have severe necrosis, hepatocytes with ballooning degeneration and collapse of the parenchyma; immunohistochemistry showed that (**B**) the expression of HBsAg was found in hepatocytes of HBV-ACLF patients and that a cohort of (**C**) CD68^+^ macrophages, (**D**) CD3^+^ T cells, and (**E**) CD16^+^ monocytes and (**F**) fibrinogen^+^ cells were infiltrated and distributed throughout the livers of HBV-ACLF patients. The arrows indicate positive cells, and the scale bar indicates 20 μm.

To address the relevance of BTLA and HVEM expression in human viral hepatitis, four liver biopsies from patients with biochemical, histological, and clinical evidence of HBV-ACLF, sixteen cases of CHB patients and five cases of healthy individuals were collected in this study. Severe hepatocyte necrosis, ballooning degeneration, and collapse of the parenchyma were found in the liver tissue from HBV-ACLF patients, as demonstrated by H&E staining (Figure 1A). In parallel, a cohort of HBs^+^ hepatocytes (Figure 1B), infiltrating CD68^+^ macrophages (Figure 1C), CD3^+^ T cells (Figure 1D), CD16+ monocytes (Figure 1E) and a high level of fibrinogen deposited in the damaged hepatocytes (Figure 1F) were also detected in these samples. Based on the histological and immunophenotypic findings from the examined samples, we concluded that these four cases were accurately diagnosed as typical instances of ACLF.

### The expression and anatomical distribution of BTLA in sections from HBV-ACLF

**Figure 2:**
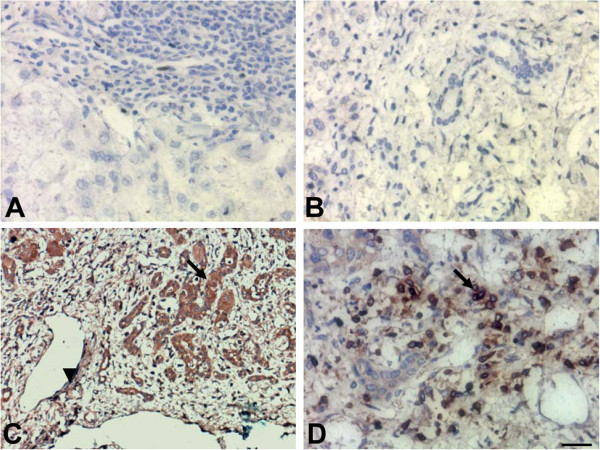
**The expression of BTLA in liver tissues from HBV-related acute-on-chronic liver failure (HBV-ACLF) patients, as detected by immunohistochemistry.** (**A**) BTLA expression was absent in normal liver sections; (**B**) BTLA expression was absent in liver sections from CHB; (**C**) strong BTLA staining was observed in benign bile ducts sections from HBV-ACLF patients; (**D**) strong BTLA staining was observed on infiltrating lymphocytes of sections from HBV-ACLF patients. The arrows indicate infiltrating positive cells, arrowhead indicated the capillaries. The scale bar indicates 20 μm.

The expression of BTLA in liver samples from HBV-ACLF patients, CHB patients and healthy individuals was detected by immunohistochemistry. The results showed that BTLA was absent in normal liver tissues (Figure 2A) and in the 16 samples from CHB patients (Figure 2B). In addition, no positive staining was observed in the sections incubated with rabbit IgG1 isotype antibodies (data not shown). Nevertheless, strongly BTLA^+^ cells were observed in the benign bile ducts (Figure 2C) and in the infiltrating inflammatory cells (Figure 2D) of these four cases of HBV-ACLF.

**Figure 3:**
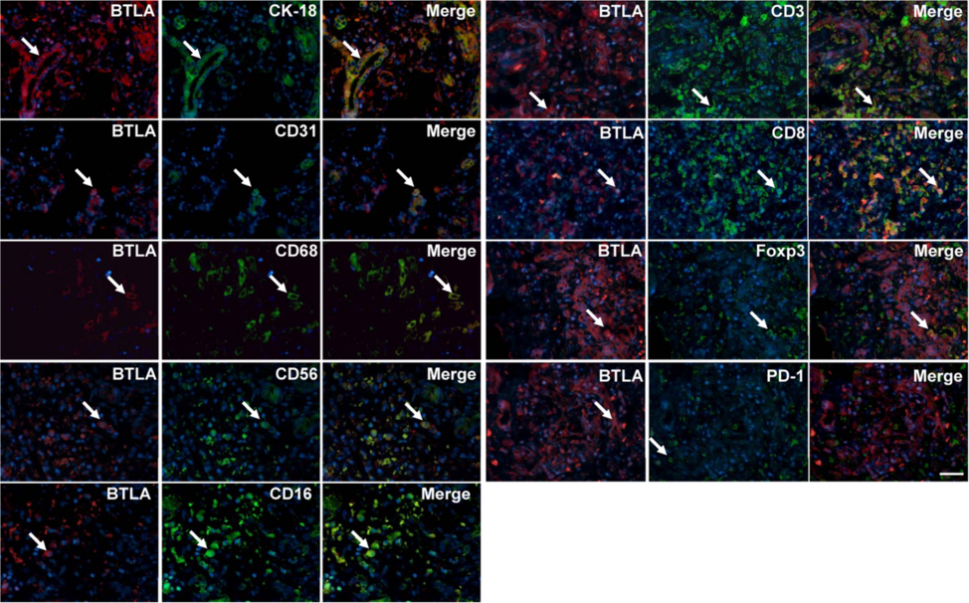
**The morphology of BTL**A^+^ c**ells in liver tissues from patients with HBV-related acute-on-chronic liver failure (HBV-ACLF) was detected by immunofluorescence double staining.** Immunofluorescence double staining revealed that BTLA was expressed on CK-18^+^ epithelial cells, CD31^+^ endothelial cells, CD68^+^ macrophages, CD56^+^ NK cells, CD16^+^ monocytes, CD3^+^ CD8^+^ T cells, and Foxp3^+^ regulatory T cells (Treg), whereas BLTA was absent on PD-1^+^ cells. The arrow indicates positive cells. The nuclei were stained with DAPI, and the scale bar indicates 20 μm.

The phenotypes of BTLA^+^ cells in liver tissues from HBV-ACLF patients were further examined by immunofluorescence double staining. From these analyses, the expression of BTLA was observed on CK-18^+^ epithelial cells, CD31^+^ endothelial cells, CD68^+^ macrophages, CD56^+^ NK cells, CD16^+^ monocytes, CD3^+^ CD8^+^ T cells, and Foxp3^+^ regulatory T cells (Treg), but this expression was completely absent on PD-1^+^ cells (Figure 3).

### The expression and distribution of HVEM in sections from HBV-ACLF

**Figure 4:**
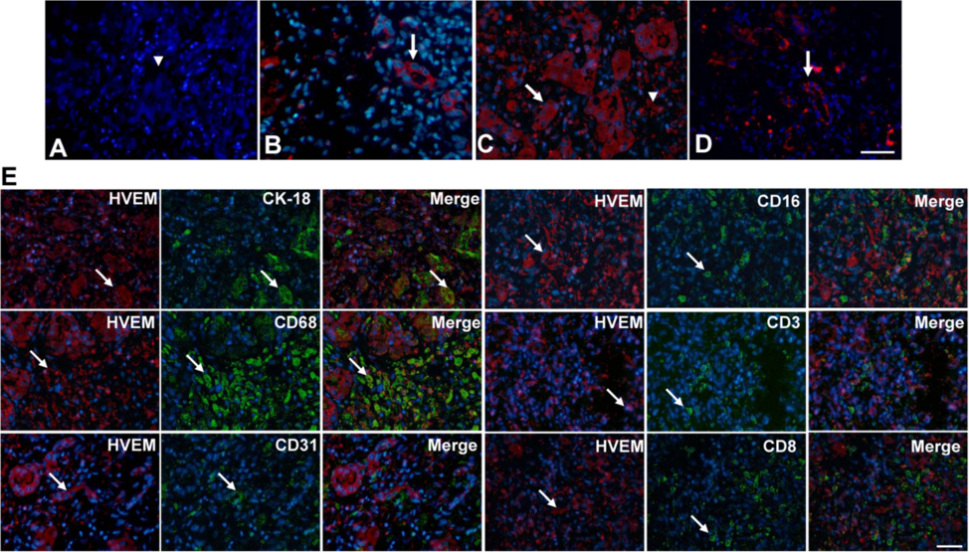
**The expression of HVEM in liver tissues from patients with HBV-related acute-on-chronic liver failure (HBV-ACLF), as detected by immunofluorescence staining.** (**A**) HVEM was absence on sections from normal individuals. The bile ducts (**B**) and infiltrating lymphocytes (**C**) (indicated by arrowhead) of liver sections from HBV-ACLF patients are positive for HVEM. (**D**) The expression of HVEM in bile ducts was also found in two cases of CHB. **(E)** Immunofluorescence double staining demonstrated that HVEM was expressed on CK-18^+^ epithelial cells and CD68^+^macrophages, whereas it was absent on CD3^+^ T cells, CD8^+^ T cells, CD16^+^ monocytes, and CD31^+^ endothelial cells. The arrows indicate positive cells. The nuclei were stained with DAPI, and the scale bar indicates 20 μm.

The expression of HVEM in these collected liver samples was detected by immunofluorescence staining, which indicated that HVEM-positive cells were observed in all of the liver samples from HBV-ACLF patients. This protein was found on cell membranes and in the cytoplasm, and its expression was chiefly found in damaged bile ducts (Figure 4B) as well as in certain infiltrating inflammatory cells (Figure 4C), which were distributed throughout the entire tissue section. In this study, two cases (2/16) of CHB were positive for HVEM, and the expression of HVEM in these instances was restricted to the damaged bile ducts (Figure 4D). However, normal liver tissues were negative for HVEM (Figure 4A). These results indicate that the expression of HVEM was induced in liver samples from HBV-ACLF patients.

The phenotypes of HVEM^+^-cells within HBV-ACLF liver sections were further examined by immunofluorescence double staining. The results showed that the expression of HVEM was found on CK-18^+^ epithelial cells and CD68^+^ macrophages, but this expression was absent on CD31^+^ endothelial cells, CD16^+^ monocytes, and CD3^+^ CD8^+^ T cells (Figure 4E).

**Figure 5:**
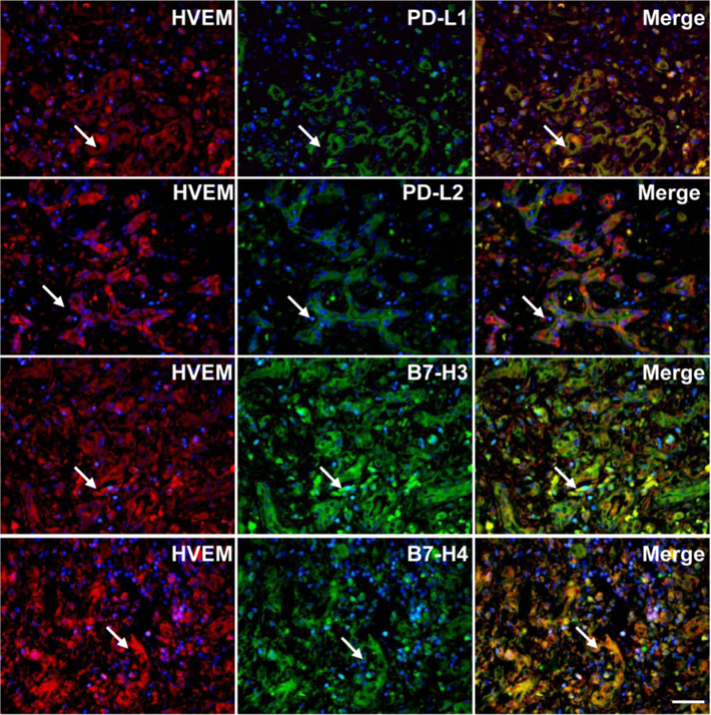
**The relationship between the expression of BTLA/HVEM and certain members of the B7 superfamily in liver tissue sections from patients with HBV-related acute-on-chronic liver failure (HBV-ACLF).** Immunofluorescence double staining indicated that several novel members of the B7 superfamily, including PD-L1, PD-L2, B7-H3 and B7-H4, were co-expressed with HVEM. The arrows indicate positive cells. The nuclei were stained with DAPI, and the scale bar indicates 20 μm.

Our previous work demonstrated that the expression of several members of the B7 superfamily, including PD-L1 (B7-H1), PD-L2 (B7-DC), B7-H3 and B7-H4, was also detected in liver biopsies from HBV-ACLF patients [14,15]. In the current study, we examined the relationships between HVEM and these members of the B7 superfamily, and the results of this analysis indicated that HVEM was co-expressed with all of these molecules in liver sections from HBV-ACLF patients (Figure 5).

### The relationships between BTLA-HVEM and FGL2 in sections from HBV-ACLF

**Figure 6:**
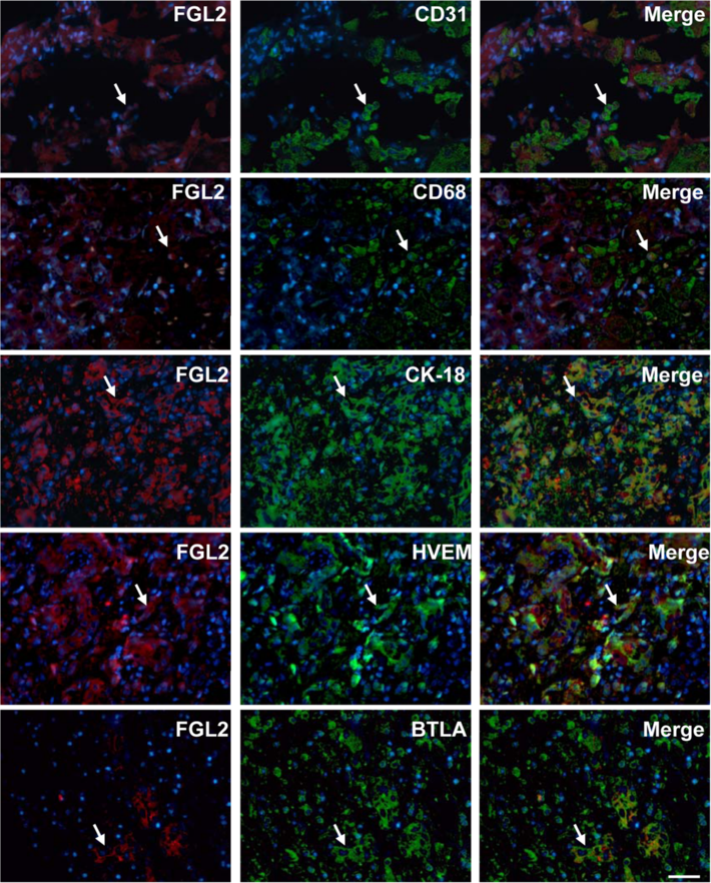
**The relationship between BTLA-HVEM and fibrinogen-like protein 2 (FGL2) expression in sections from patients with HBV-related acute-on-chronic liver failure (HBV-ACLF).** Immunofluorescence double staining demonstrated that FGL2 expression was observed in CD31^+^ endothelial cells, CD68^+^ macrophages, and CK-18^+^ bile ducts of HBV-ACLF patients. Moreover, BTLA and HVEM were co-expressed with FGL2. The arrows indicate positive cells. The nuclei were stained with DAPI, and the scale bar indicates 20 μm.

The hallmark of ACLF is the presence of sinusoidal thrombosis and associated hepatocellular necrosis, these phenomena are accompanied by the expression of virus-induced FGL2 in the cells of the sinusoidal lining. The FGL2 protein has the capacity to activate the coagulation cascades directly, a function that has been described as procoagulant activity (PCA) [4]. In this study, FGL2 expression was found in these HBV-ACLF samples, and immunofluorescence double staining indicated that CK-18^+^ epithelial cells, CD68^+^ macrophages and CD31^+^ endothelial cells are the major source of this protein. The expression of BTLA/HVEM and FGL2 in HBV-ACLF sections was further measured. The results demonstrated that both BTLA and HVEM were co-expressed with FGL2 (Figure 6). Our combined results revealed that both BTLA and HVEM were enriched in HBV-ACLF patients, suggesting that these molecules can be used as biomarkers for the pathological diagnosis of HBV-ACLF.

## Discussion

The BTLA/HVEM inhibitory pathway appears to be particularly important for balancing immune response and tolerance in autoimmune diseases, tumor development, transplantation, allergy and, in particular, the pathogenesis of viral infection [16-18]. Recent studies have suggested that the BTLA/HVEM interaction functionally regulates CMV-specific T cell functions [9]. Our previous work has also demonstrated that BTLA signaling can maintain the survival of macrophages that are infected with MHV-3, resulting in virus distribution and tissue damage following MHV-3 infection [13]. **ACLF is a severe clinical syndrome characterized by coagulopathy, jaundice and hepatic encephalopathy **[19,20]. **Most ACLF cases in China are associated with HBV infection.** In this study, we extended our research and examined the expression of BTLA/HVEM in four cases of HBV-ACLF patients. Immunohistochemical analysis revealed that the expression of BTLA was dramatically enhanced in liver samples from these HBV-ACLF patients, and the positive cells were distributed throughout the entire tissue. However, no positive cells were observed in the liver tissue of CHB patients or healthy individuals. The phenotypes of BTLA^+^ cells were further analyzed by immunofluorescence double staining, and the results demonstrated that BTLA was expressed on CK-18^+^ epithelial cells, CD31^+^ endothelial cells, CD68^+^ macrophages, CD56^+^ NK cells, CD16^+^ monocytes, CD3^+^ CD8^+^ T cells, and Foxp3^+^ regulatory T cells (Tregs). **Although the limitation of HBV-ACLF cases was included here, the expression of BTLA was observed on all of these four cases and these data strongly** indicates that BTLA may actively participate in regulating the pathogenesis of this disease.

HVEM, the ligand of BTLA, is a novel member of the TNF receptor superfamily, and its mRNA is broadly expressed in both lymphoid and non-lymphoid tissues [18]. The expression and distribution of HVEM in liver biopsies from HBV and HBV-ACLF patients has not previously been reported. In this study, our data indicated that HVEM was found in sections from HBV-ACLF and was restricted to the damaged bile ducts and certain infiltrating cells, whereas HVEM was absent in sections from normal individuals. Assessments using immunofluorescence double staining further determined that HVEM was expressed on CK-18^+^ epithelial cells and CD68^+^ macrophages but completely absent on CD31^+^ endothelial cells, CD16^+^ monocytes, and CD3^+^ CD8^+^ T cells. These results demonstrated that HVEM was induced in certain antigen-presenting cells (APCs) in these patients.

Our previous work has revealed that the expression of several members of the B7 superfamily, including **PD-L1 (B7-H1), PD-L2 (B7-DC),** B7-H3 and B7-H4, was found in liver sections from HBV-ACLF [14,15]. **PD-L1 and PD-L2** are two immunoregulatory molecules belonging to the B7 superfamily that were identified as ligands for PD-1. The binding of **PD-L1 or PD-L2** with PD-1 activates a signaling pathway that inhibits T cell responses [21,22]. B7-H3 is a type I transmembrane protein that is expressed on activated macrophages, dendritic cells, monocytes and several types of cells in non-lymphoid tissues [23]. B7-H3 promotes the proliferation, cytotoxicity and IFN-γ production of T cells by crosslinking with its hypothetical receptor and triggering the receptor-mediated expression of myeloid cell-like transcript 2 (TLT-2) on activated T cells [23,24]. B7-H4 is a GPI-linked protein, and mRNA encoding B7-H4 is widely distributed in murine and human peripheral tissues [25,26]. B7-H4^−/−^ mice exhibited augmented Th1 responses and displayed lowered parasite burdens upon *Leishmania major* and *Listeria monocytogenes* infection compared with wild type mice, suggesting an inhibitory role for B7-H4 in T cell responses [27,28]. In this study, we also detected the relationships between HVEM and these members of the B7 superfamily in sections from HBV-ACLF, and the study results demonstrated that HVEM was co-expressed with all of these molecules.

FGL2 is a critical molecule that promotes fibrinogen deposition, which, in turn, activates the coagulation cascades and thereby induces PCA, causing hepatocellular necrosis during the development of fulminant virus hepatitis [2]. High levels of FGL2^+^ cells that are distributed throughout the liver have also been observed in HBV-ALCF patients. Phenotypic analyses indicated that the expression of FGL2 was mainly localized to infiltrating CD68^+^ macrophages, CK-18^+^ bile ducts, and CD31^+^ capillaries. Moreover, both BTLA and HVEM were co-expressed with FGL2, as assessed by immunofluorescence double staining.

To the best of our knowledge, this report is the first to investigate the expression of BTLA and its ligand, HVEM, in liver sections from HBV-ACLF patients. Our results indicate that these molecules might be useful diagnostic biomarkers for HBV-ACLF, and an understanding of the functional roles of these molecules could aid in the development of novel strategies for disease diagnosis or immunotherapy.

## Competing interests

None of the authors have any conflicts of interest related to this manuscript.

## Authors’ contributions

HX: Immunohistochemistry; DC: Immunofluoresence double staining; GG: Samples collections and H&E staining; ZR: Immunofluoresence double staining; YW: Revised the manuscript and experimental design; YC: Writing this manuscript and experimental design. All authors read and approved the final manuscript.
